# A visualized and scientometric analysis of research trends of weight loss in overweight/obese children and adolescents (1958–2021)

**DOI:** 10.3389/fpubh.2022.928720

**Published:** 2022-10-20

**Authors:** Guotao Sun, Long Li, Xiaolin Zhang

**Affiliations:** ^1^School of Physical Education, Chengdu Sport University, Chengdu, China; ^2^College of Education and Sports Science, Yangtze University, Jingzhou, China; ^3^School of Sports Medicine and Health, Chengdu Sport University, Chengdu, China; ^4^School of Physical Education, Xichang University, Xichang, China; ^5^Sport Research Office, Chengdu Sport University, Chengdu, China

**Keywords:** weight loss, children and adolescents, overweight/obese, scientometric analysis, visualized analysis, Citespace, mapping knowledge domains

## Abstract

**Background:**

Weight loss is an appropriate approach to reduce the health risks associated with overweight/obese children and adolescents, and the optimal method of weight loss requires further research. This study systematically explores scientific co-operation, disciplinary interaction, hotspots and trends in the field of weight loss in overweight/obese children and adolescents (WLOCA), and provides references for further research.

**Methods:**

Citespace 5.8.R1 (64-bit) was adopted to conduct a comprehensive visualization analysis of the literature on WLOCA from Web of Science Core Collection, including publication, institution, country/region, author, journal, keywords and reference.

**Results:**

2,513 papers were found in the Web of Science Core Collection, and the annual number of papers published has increased significantly since 2003. Cincinnati Children's Hospital is the institution with the largest number of publications, while Washington University plays a pivotal role in the collaboration network. In terms of nations, USA has made greater contributions than the rest in terms of the number of publications and global co-operation research. The most influential authors in this field are Thomas H. Inge, Thomas Reinehr, Todd M. Jenkins, Epstein LH, Ogden CL, etc. The most active journals are “*Obesity*,” “*International Journal of Obesity*,” “*Obesity Surgery*,” “*Pediatrics*,” etc. which are characterized by interdisciplinary interactions. Research hot topics mainly include “assessment of obesity and pathophysiological mechanism,” “comprehensive intervention,” and “bariatric surgery,” and there's a gradual shift from “lifestyle intervention” and “pathophysiological mechanism” to “clinical surgical application.” In addition, disciplinary integration and comprehensive research, targeted intervention and treatment, and prospective research are the future research trends.

**Conclusion:**

The overall trend in WLOCA study is positive. The main contribution of this study is to reveal the overall picture of the research in this field with visual maps and detailed data by combining quantitative with qualitative approaches, which can provide valuable references for relevant researchers to quickly understand the status of studies on WLOCA, to seek co-operation, and grasp research hotspots and frontier trends.

## Introduction

Overweight/obesity in children and adolescents is one of the most important public health problems. Globally, the total prevalence rate of overweight/obesity increased by 27.5% in adults and 47.1% in children during 1980–2013 (1). Over the past 40 years, the rising trend of overweight/obesity among children and adolescents has leveled off in some high-income countries, but its proportion is still very high. Meanwhile, rates of overweight/obesity are accelerating in some Asian countries (2, 3). According to WHO, the number of overweight/obese children and adolescents aged 5–19 years exceeded 340 million in 2016, and 39 million children under the age of 5 were overweight or obese in 2020 (4). It is worrying that the global spread of COVID-19 has further increased the number of obese children since 2019 (5, 6).

Overweight/obesity in children and adolescents are associated with higher risk and earlier onset of chronic diseases such as T2DM and cardiovascular (CV) disease (7, 8), which may lead to lifelong overweight/obesity (9), with adverse psychosocial consequences and lowered education (10, 11), and has great negative impacts on society and economy (12). To make it worse, obesity may lead to earlier puberty in children (13), irregular menstruation in adolescent girls (14), and obstructive sleep apnea syndrome (OSAS) (15). Further research found that OSAS is closely related to CV disease, chronic neurodegenerative diseases, and inflammatory diseases, leading to a high risk of cognitive impairment (16). Obesity also contributes to childhood CV disease and impairs autonomic function in children and adolescents, with reduced vagal or HRV parasympathetic activity being the most common, along with concurrent reductions in sympathetic activity and reduced baroreflex sensitivity (17). Interaction between obesity and OSAS increases CV morbidity in children (18), and overweight in adolescence leads to additional CV events (19).

As early as 2002, overweight/obesity in children and adolescents has been ranked by WHO as one of the 10 leading global risks for burden of disease. In 2014, WHO established the “Ending Childhood Obesity Committee,” which proposed several key behaviors to end childhood obesity. Countries around the world have also taken action to address overweight/obesity in children and adolescents. For example, in 2017, the American Endocrine Society updated its practical guidelines on prevention, assessment and treatment of childhood obesity (20). Studies have also found that WLOCA is effective in reducing the risk of various diseases and significantly improving weight-related quality of life (21–24). Over the past few decades, researchers have conducted extensive studies on how to effectively reduce the weight of overweight/obese children and adolescents. Several review articles generally focus on the effectiveness of weight loss, such as motivation (25), behavior (26), exercise (27, 28), and comprehensive intervention of combined nutrition (29). Although there are certain risks, drug combined behavioral therapy (30, 31) and bariatric surgery (32, 33) have also achieved good weight loss and improved quality of life. Some systematic reviews have also reviewed the efficacy of different interventions and treatments (34–36). However, facing the current challenge of high numbers of overweight/obese children and adolescents, the best way to weight loss has yet to be found. Traditional research mostly focuses on the discussion of local knowledge or qualitative analysis on the whole, and cannot quantitatively analyze the progress of knowledge in a specific field from a large number of literatures, such as scientific co-operation, disciplinary interaction, research hotspots and trends, etc., which is not conducive to researchers' quick understanding of the current research status in the field. In order to promote the further development of the research, it is necessary to use scientific research tools to comprehensively and intuitively reveal the characteristics and rules of the existing research. Therefore, there is a strong need for scientometric and visualization analysis of research trends of WLOCA.

Scientometrics is a science that applies statistical and computational techniques to quantitatively analyze the inputs, outputs, and processes of scientific activities so as to find out the corresponding laws. Citespace is an information visualization analysis tool developed under the background of scientometrics and data visualization, which can visually analyze the network, structure, interaction, crossover and evolution of knowledge units (37, 38). In recent years, it has been widely adopted not only in information science, but also in such disciplines as medicine (39–41), public health (42–44), artificial intelligence (45, 46), environmental ecology (47), and educational science (48). Therefore, this paper adopts Citespace to systematically investigate the literature on WLOCA, attempting to identify the potential and valuable information for further research from a new perspective, and provides references for the formulation or improvement of relevant policies for the prevention and treatment of overweight/obesity in children and adolescents.

This paper addresses the following questions: (i) How did the researcher' concerns change in this field? (ii) Which research institutions, countries/regions, scholars, journals, subject categories and references play important roles in this field? (iii) What are the research hotspots and frontier trend?

## Materials and methods

### Data source and retrieval

All data used in this study were obtained from the Web of Science Core Collection (WoSCC), indexed by SCI-EXPANDED (1900-present), SSCI (1998-present), A&HCI (1998-present), CPCI-S (1998-present), CPCI-SSH (1998-present), ESCI (2015-present), CCR-EXPANDED (1985-present), and Index Chemicus (1993-present). WoSCC is adopted because it contains a comprehensive indexing record of many influential and high-quality journals around the world. At the same time, WoSCC is more comprehensive than PubMed, which only focuses on biomedical fields (49). Compared with Scopus, the databases covered by the two have little difference (43). Scopus did not have references before 1996 (50), which is not convenient for overall retrieval. Moreover, it has been shown that Knowledge mapping drawn by WoSCC articles is more desirable compared to other databases when using Citespace for visualization analysis (51). In addition, WoSCC has been used as the only data source in numerous previous scientometric and visualization studies (52–56).

**Figure 1:**
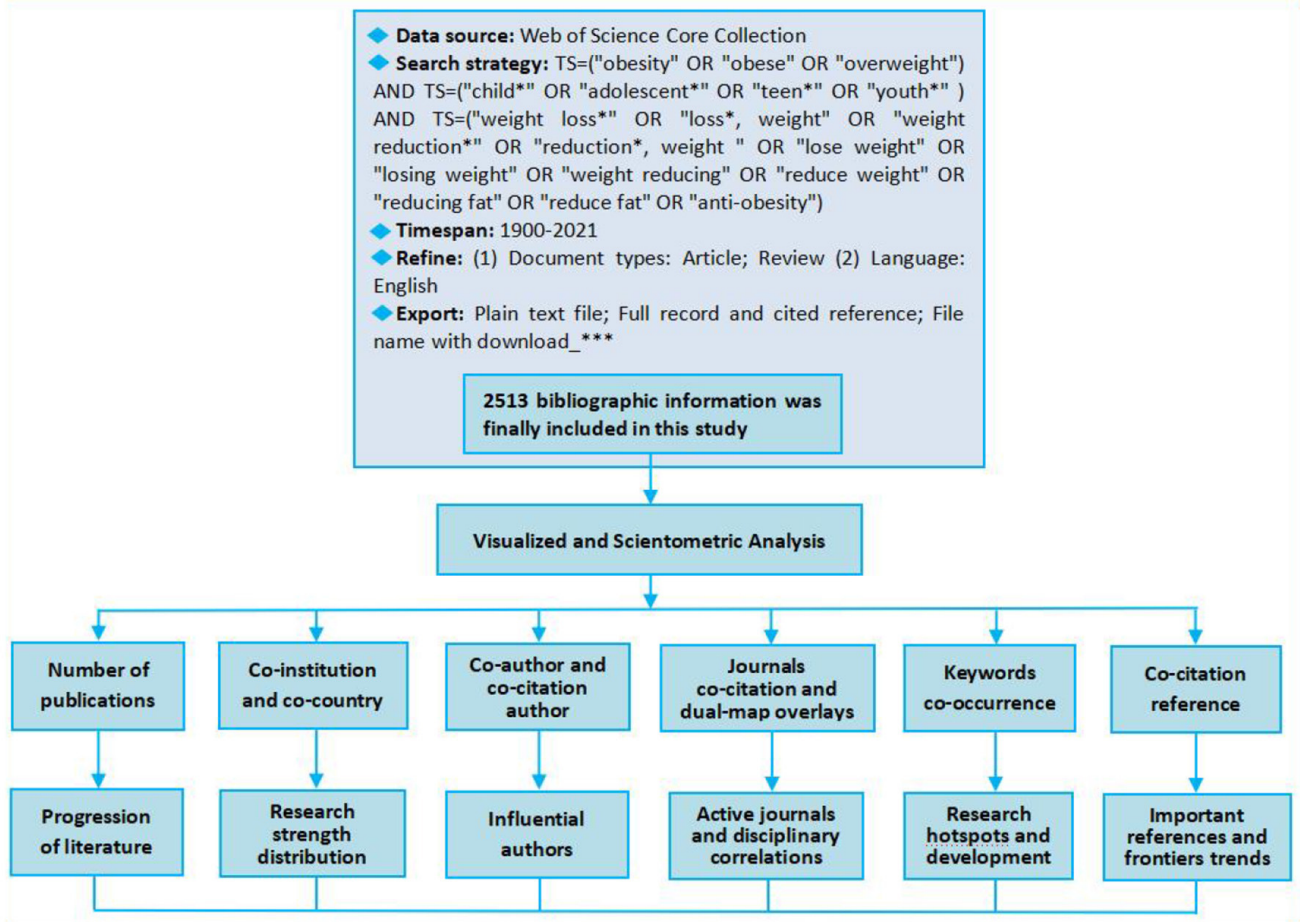
Design of the study.

The data retrieval process is as follows: First, all the search terms of overweight/obese, children/adolescents, and weight loss are determined respectively through MeSH and entry terms, and the synonyms of “weight loss” in the literature are selected for combined retrieval to ensure a comprehensive search. Second, a multi-topic retrieval was conducted: TS = (“obesity” OR “obese” OR “overweight”) AND TS = (“child^*^” OR “adolescent^*^” OR “teen^*^” OR “youth^*^”) AND TS = (“weight loss^*^” OR “loss^*^, weight” OR “weight reduction^*^” OR “reduction^*^, weight” OR “lose weight” OR “losing weight” OR “weight reducing” OR “reduce weight” OR “reducing fat” OR “reduce fat” OR “anti-obesity”), time spans = 1900–2021, document types = article and review, language = English. Finally, the relevance of the retrieved documents was identified one by one through the title and abstract, irrelevant documents were eliminated, and the bibliographic information of 2,513 documents that met the requirements was downloaded according to the corresponding format of the analysis software (The retrieval time is December 31, 2021). There are two main reasons for the exclusion: first, the research subjects must be overweight/obese children and adolescents (excluding athletes) and their related problems; second, the research content directly points to overweight/obesity, not just mentioned in the background or suggestions of the abstract. The overall research design of this paper is shown in Figure 1.

### Methods

Citespace 5.8.R1 (64-bit), the analysis tool adopted in this paper, is a free visualization analysis software developed by Dr. Chaomei Chen of Drexel University, USA. The visualization and analysis methods mainly include co-institution and co-country/region, co-author and co-citation author, co-citation journal, dual-map overlays, keyword co-occurrence, co-citation reference, and bursts detection. The parameters in the specific operation of Citespace in this paper are as follows: the time span is 1958–2021, because the first literature retrieved was published in 1958, and the slice length = 2. In each time slice the top 25 nodes were selected for keyword co-occurrence, the top 20 nodes for reference co-citation, and the top 30 nodes for the rest. The network pruning used the pathfinder except for the minimum spanning tree (MST) for keyword co-occurrence and literature co-citation.

In Citespace, Modularity Q and Mean Silhouette (S) are the basis of the map rendering effect. Q > 0.3 means that the network structure is significant, S > 0.5 indicates that the clustering is reasonable, and S > 0.7 suggests that the clustering is highly convincing (57). Therefore, in order to meet the above requirements, we finally determined the parameters and algorithms by repeatedly debugging them. At the same time, in view of the partial overlap of some nodes in the co-occurrence and co-citation knowledge graph, we moved them appropriately without changing the network structure to ensure the best visual presentation effect. The nodes in the knowledge graph are the actual knowledge units analyzed, corresponding to institution, country/region, author, journal, category, keyword, and reference, and their size corresponds to how often the nodes appear or are cited, while the color represents the historical chronology of their appearances or citations, and the nodes marked with purple indicate their greater centrality. The thickness of the connecting line indicates the affinity between nodes, and the color of the connecting line represents the time of first co-occurrence or co-citation, from light to dark corresponding to the time from far to near, and the higher centrality indicates that the node is more important in the network (58).

## Results

### Visualization and analysis on publication outputs

**Figure 2:**
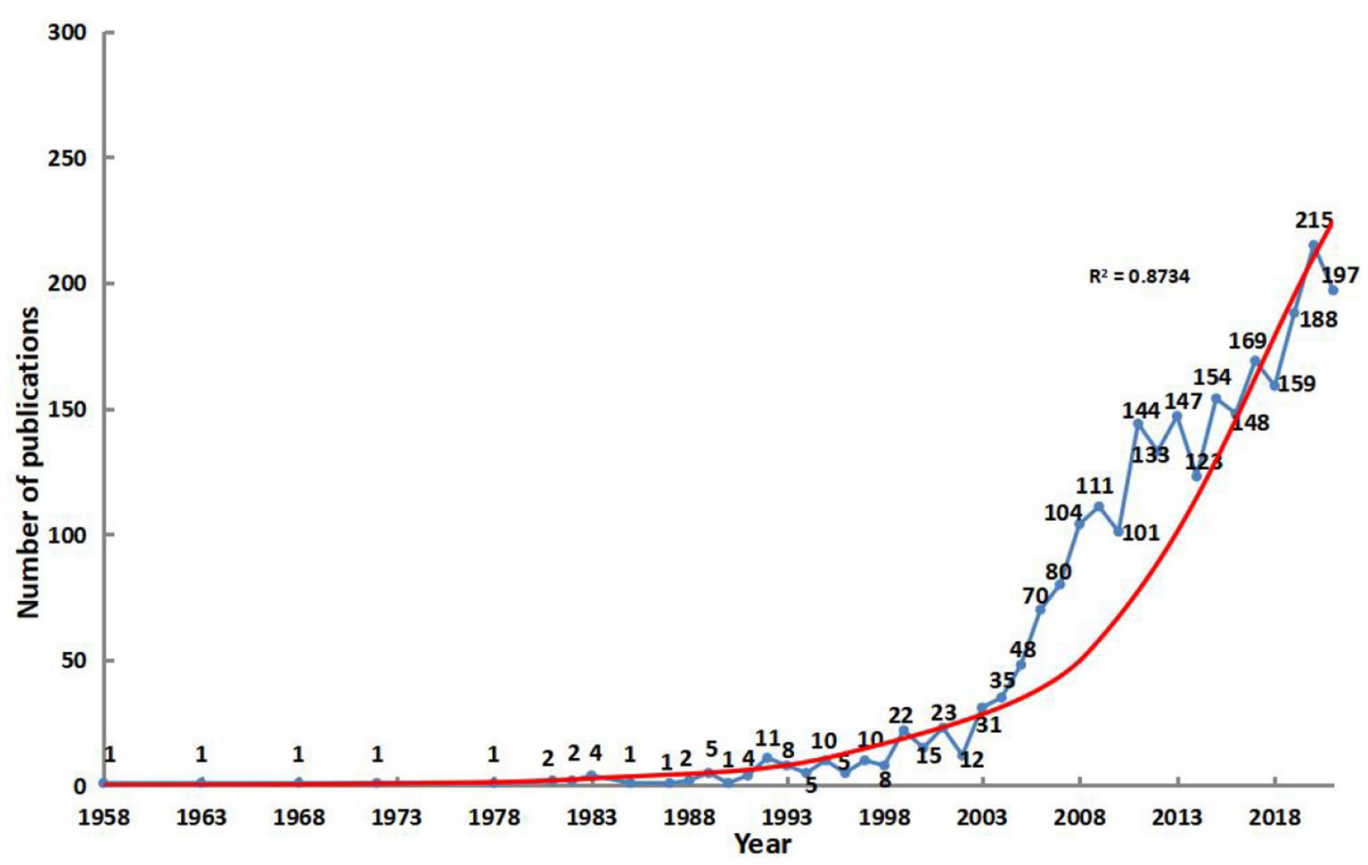
Literature on WLOCA published from 1958–2021.

To some extent, the numbers of publications reflect the changes in researchers' concerns in a specific field. Figure 2 shows that the papers on WLOCA were few and far between before 2002. After a period of sustained growth, it exceeded 100 for the first time in 2008, and then fluctuated but maintained an overall increasing trend, reaching a maximum of 215 in 2020. The trend forecasting model for the number of papers on WLOCA (*R*^2^ = 0.8734) indicates that this field is receiving increasing attention and the related research will be on the rise.

### Visualization and analysis on co-institutions and co-countries/regions

**Figure 3:**
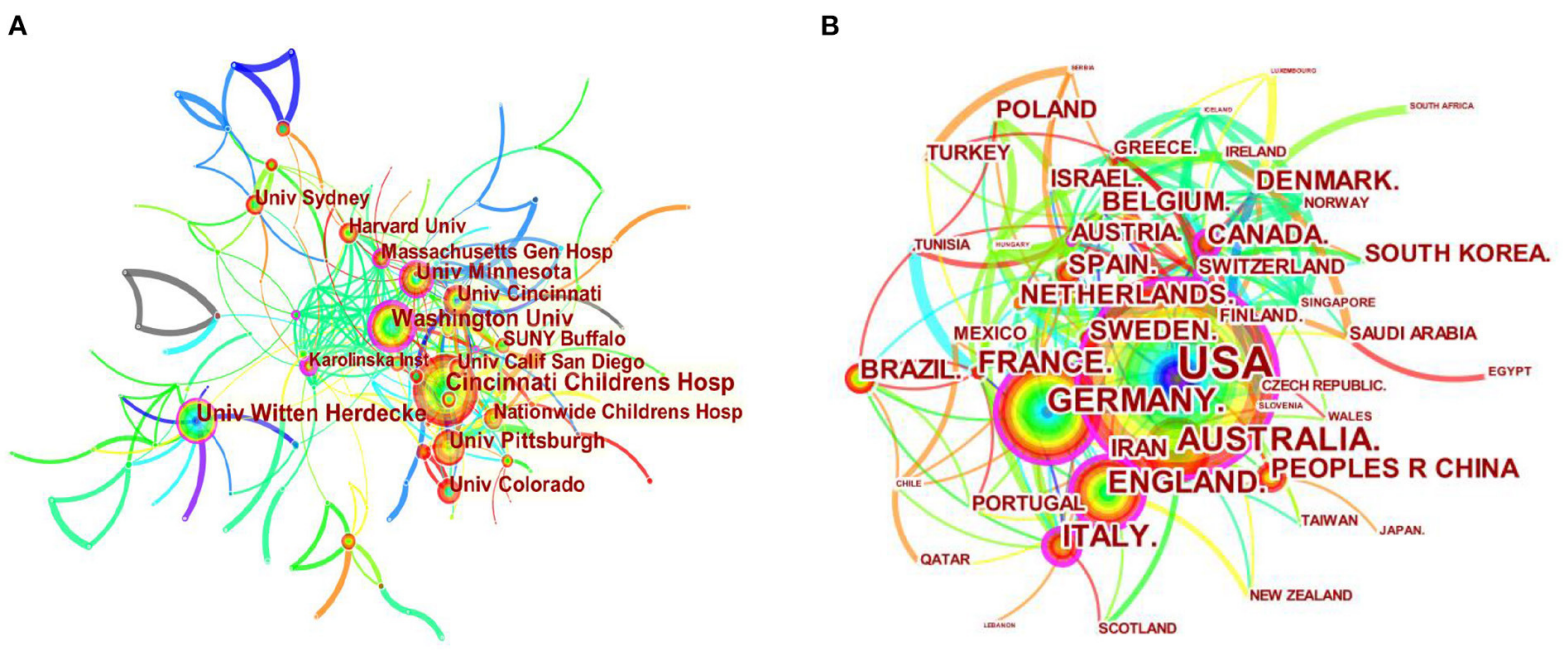
The institution collaboration network in the research of WLOCA **(A)**; Co-country/region distribution network in the research of WLOCA **(B)**.

Collaboration across institutions has always been common, expected, necessary, and valued in academic research. As shown in Figure 3, 174 institutions and 52 countries/regions, respectively, participated in collaborative research on weight loss of children and adolescents, with an institutional collaboration network density of 0.0224 and a national or regional collaborative network density of 0.1403, the latter being relatively close. Details of the top 10 institutions and countries/regions are shown in Table 1. All are universities except a children's hospital, and seven of the universities are in the United States. Cincinnati Children's Hospital ranks the first place with 105 papers in terms of the number of papers published. The top three institutions in terms of centrality are Washington University (0.22), University of Minnesota (0.18) and the University of Witten Herdecke (0.14). As for the country/region distributions, USA ranks the first place with 1,123 papers published, followed by Germany, Australia, England, Italy, France and etc. Meanwhile, the centrality reveals that USA/England (0.17), Italy/Canada (0.14) and Germany (0.11)play key roles in the collaboration. Among the top 10 countries, only Brazil and China are developing countries.

**Table 1:** Top 10 contributing institutions by countries/regions and centrality.

**Rank**	**Institution**	**Publications**	**Centrality**	**Country**	**Publications**	**Centrality**
1	Cincinnati Children's Hosp (USA)	105	0.04	USA	1,123	0.17
2	Washington Univ(USA)	78	0.22	Germany	196	0.11
3	Univ Witten Herdecke(Germany)	74	0.14	Australia	150	0.06
4	Univ Minnesota (USA)	64	0.18	England	146	0.17
5	Univ Pittsburgh (USA)	59	0.02	Italy	116	0.14
6	Univ Cincinnati (USA)	51	0.03	France	102	0.03
7	Univ Colorado (USA)	46	0	Spain	90	0.07
8	Univ Sydney (Australia)	42	0.05	Brazil	90	0.01
9	Harvard Univ (USA)	41	0.08	Canada	87	0.14
10	Univ Calif San Diego (USA)	37	0.01	China	81	0.09

### Visualization and analysis on co-authors and co-citation authors

**Figure 4:**
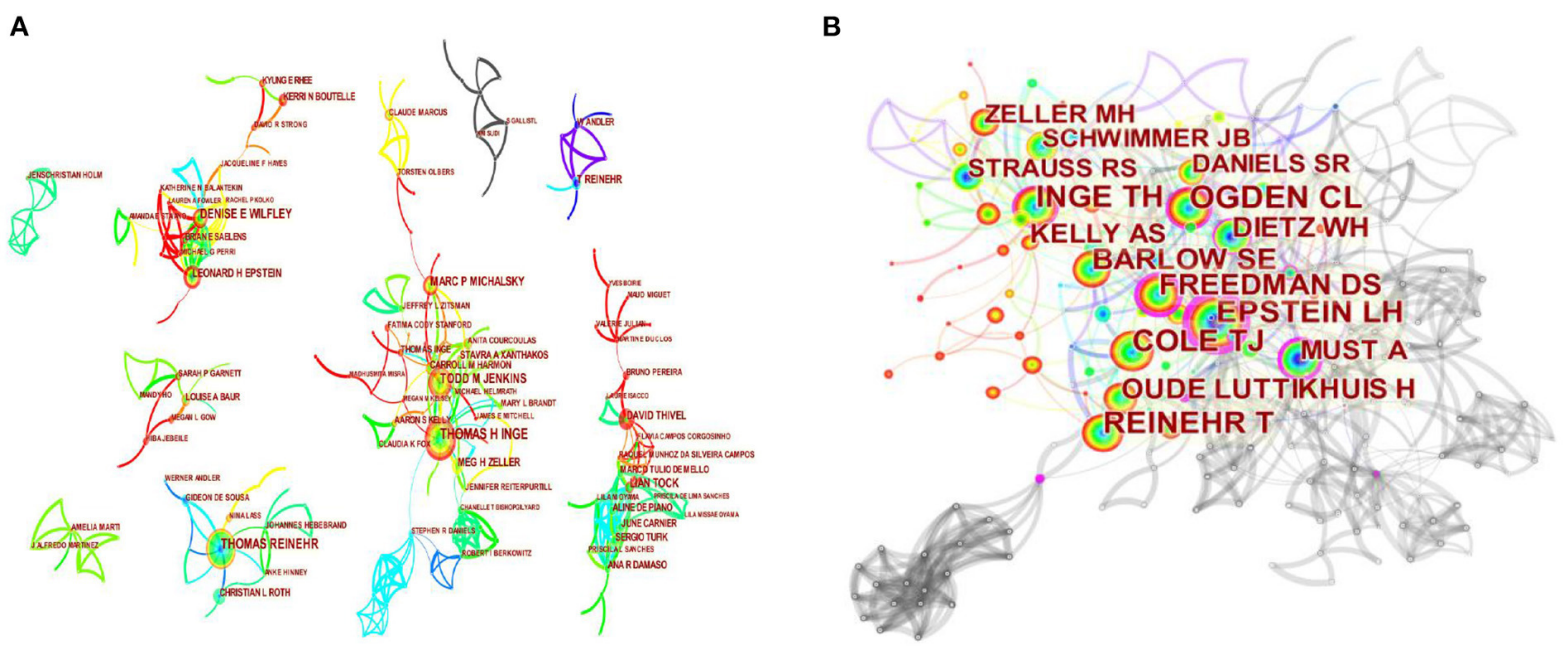
Co-author network in the research of WLOCA **(A)**; Co-citation author network in the research of WLOCA **(B)**.

Figure 4A shows that there are 428 nodes and 684 lines forming the co-author network, while the density is only 0.0075. However, only the nine largest co-author networks are shown in the figure, with Thomas H. Inge, Thomas Reinehr, Denise E. Wilfley, and Lian Tock representing the four relatively large academic communities. The top 10 authors in terms of publications are shown in Table 2, with Thomas H. Inge (68 publications) and Thomas Reinehr (55 publications) in the lead and only five authors with some centrality. Figure 4B shows that the author co-citation network consist of 263 nodes and 751 links with density of 0.0218. The citation frequencies of the top 10 co-cited authors are shown in Table 2, with Ogden CL (616 citations) in the lead and Epstein LH (0.41), Freedman DS (0.29), Must A (0.23), and Inge TH (0.17) had the relatively highest centrality and their publications were focused on by researchers.

**Table 2:** Top 10 co-author and co-citation author by frequency.

**Rank**	**Co-author**	**Frequency**	**Centrality**	**Co-citation**	**Co-citation**	**Centrality**
				**author**	**frequency**	
1	Thomas H. Inge	68	0.02	Ogden CL	616	0.11
2	Thomas Reinehr	55	0	Epstein LH	471	0.41
3	Todd M. Jenkins	42	0.01	Cole TJ	462	0.07
4	Denise E. Wilfley	34	0	Inge TH	443	0.17
5	Lian Tock	28	0.01	Reinehr T	418	0.01
6	Marc P. Michalsky	28	0.01	Barlow SE	346	0.08
7	Leonard H. Epstein	27	0	Freedman DS	327	0.29
8	David Thivel	25	0	Oude Luttikhuis H	269	0.04
9	Meg H. Zeller	24	0.01	Dietz WH	246	0.14
10	T. Reinehr	23	0	Must A	197	0.23

### Visualization and analysis on co-citation journals and dual-map overlays

**Figure 5:**
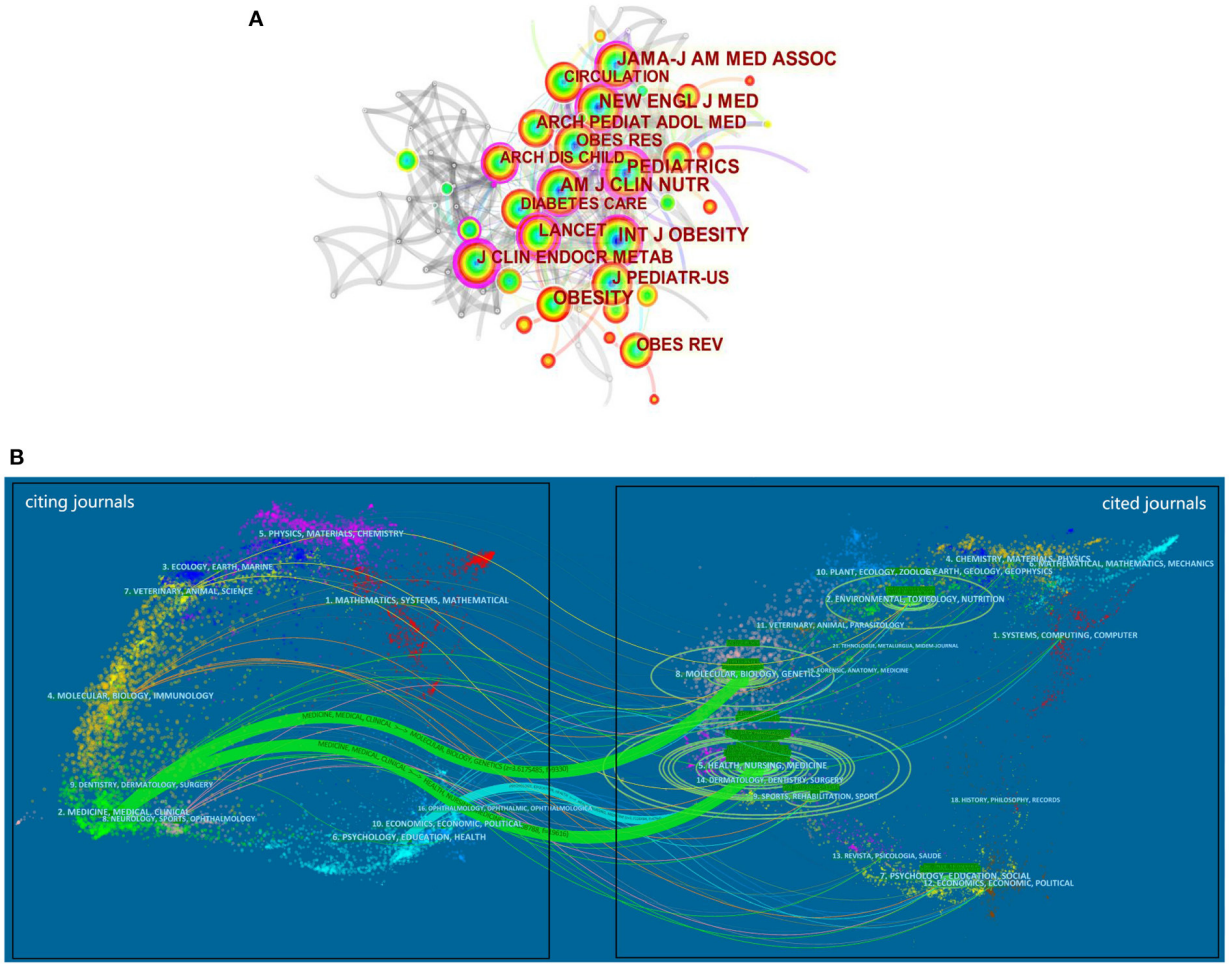
Co-citation journal network in the research of WLOCA **(A)**; A dual-map overlay of journals that published literature in the research of WLOCA **(B)**.

Active journals in this study refer to both source journals and co-citation journals. The top 10 journals are listed in Table 3. *Obesity* ranks the first place with 96 papers, followed by *International Journal of Obesity, Obesity Surgery*, and *Surgery for Obesity and Related Diseases*. The co-citation networks of journals consist of 114 nodes and 422 links, with density of 0.0655 (Figure 5A). Seven out of the top 10 journals in terms of co-citation frequency have more than 1,000 co-citations, with *International Journal of Obesity* being the highest, followed by *Pediatrics, Journal of the American Medical Association, Obesity*, etc. The top three journals in terms of centrality are *Pediatrics* (0.32), *Journal of Clinical Endocrinology & Metabolism* (0.23), and *International Journal of Obesity (0.18)*.

**Table 3:** Top 10 journals and co-citation journals by publications and co-citation frequency.

**Rank**	**Journal**	**Publications**	**Co-citation journal**	**Co-citation frequency**	**Centrality**
1	Obesity	96	*Int J Obesity*	1,695	0.18
2	International Journal of Obesity	95	*Pediatrics*	1,637	0.32
3	Obesity Surgery	80	*J Am Med Assoc*	1,328	0.17
4	Surgery for Obesity and Related Diseases	66	*Obesity*	1,237	0.05
5	Pediatric Obesity	61	*New Engl J Med*	1,187	0.12
6	Pediatrics	54	*Am J Clin Nutr*	1,110	0.14
7	Obesity Reviews	39	*J Pediatr-US*	1,005	0.1
8	Journal of Clinical Endocrinology & Metabolism	37	*J Clin Endocr Metab*	944	0.23
9	Journal of Pediatric Endocrinology & Metabolism	37	*Obes Res*	940	0.04
10	Childhood Obesity	36	*Obes Rev*	870	0.04

Disciplinary association is mainly established through the connection between the discipline of the citing document and the discipline where the cited document is located. Overlay maps in Citespace can realize the subject distribution and inter-subject association involved in the research (59). The left and right sides of Figure 5B represent the subject areas involved in the citing literature and the cited references, respectively. The connection lines represent the citation relationship. The thicker the connection, the closer the links between the subject areas. Among them, “Psychology, Education, Health” is mainly based on the knowledge of such disciplines as “Health, Nursing, Medicine”, while “Medicine, Medical, Clinical” mainly refer to the knowledge of such fields as “Molecular, Biology, Genetics” and “Health, Nursing, Medicine”. At the same time, “Neurology, Sports, Ophthalmology” and “Molecular, Biology, Immunology” are also the focus areas of citing literature, while “Environmental, Toxicology, Nutrition” “Sports, Rehabilitation, Sport”, and “Psychology, Education, Social” are also important sources of knowledge for citing literature.

### Visualization and analysis on keywords co-occurrence

**Figure 6:**
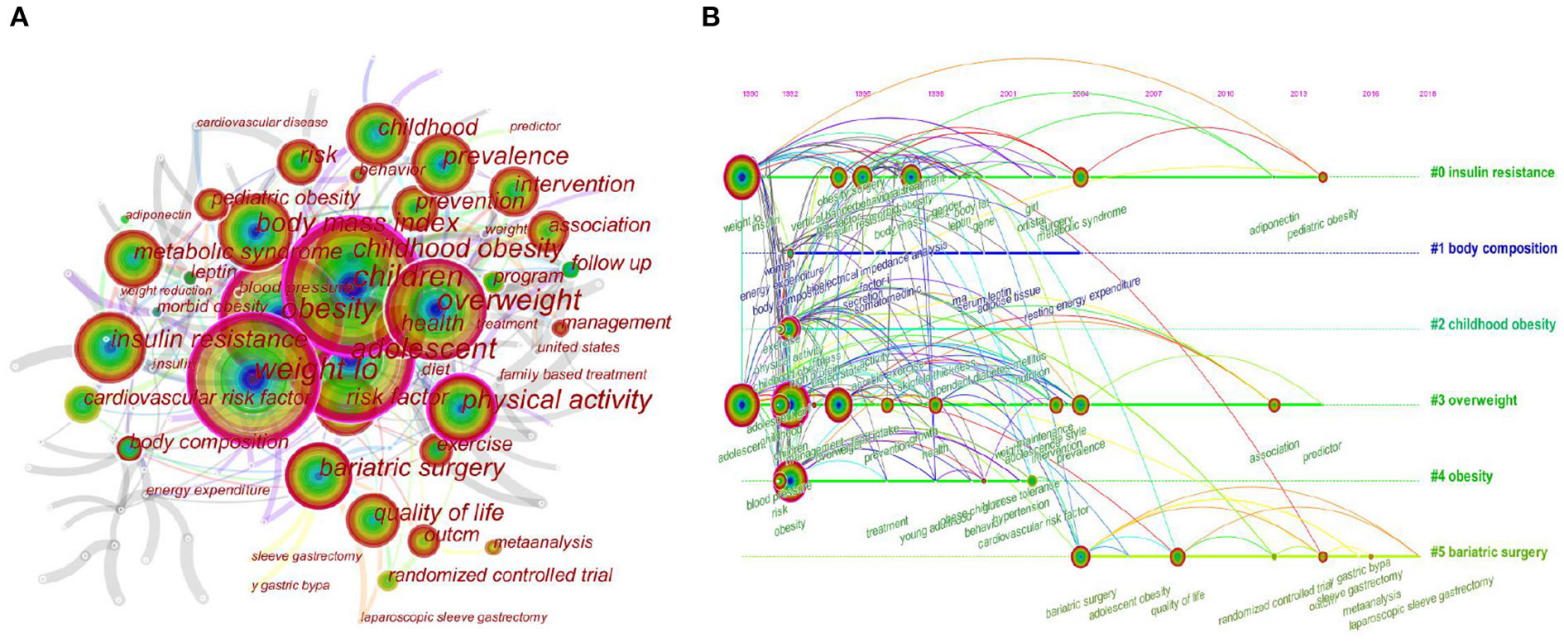
Keyword co-occurrence network in the research of WLOCA **(A)**; Timeline map of co-occurring keywords network in the research of WLOCA **(B)**.

Keywords are highly generalized and refined topics of a paper. Frequency analysis of keywords is critical for identifying the hot research topics in a given field (60). The synonymous keywords “child” were merged into “children” and “family-based treatment” into “family based treatment”. The map of keyword co-occurrence is shown in Figure 6A. The top 20 keywords in terms of frequency and their centrality are listed in Table 4. It can be seen that such keywords as “body mass index, physical activity, bariatric surgery, insulin resistance, prevalence, intervention, metabolic syndrome, health, risk factor, prevention, and quality of life” constitute the main hot topics of research in this field.

**Table 4:** Top 20 keywords by frequency.

**Rank**	**Keyword**	**Frequency**	**Centrality**	**Rank**	**Keyword**	**Frequency**	**Centrality**
1	Weight loss	1,129	0.34	11	Prevalence	272	0.06
2	Children	1,098	0.42	12	Intervention	244	0.01
3	Obesity	1,050	0.22	13	Childhood	236	0.09
4	Adolescent	960	0.21	14	Metabolic syndrome	232	0
5	Overweight	845	0.13	15	Health	213	0.11
6	Childhood obesity	509	0.15	16	Risk factor	198	0.04
7	Body mass index	501	0.09	17	Prevention	190	0.01
8	Physical activity	446	0.31	18	Quality of life	180	0.01
9	Bariatric surgery	396	0.16	19	Risk	179	0.03
10	Insulin resistance	306	0.06	20	Association	143	0

Figure 6B is a timeline map produced with clustering analysis of the keywords of the papers. There are six categories in total. The keywords representing the same kind of research content are presented on the same line in chronological order. The right tags are the most representative keywords extracted by Log-likelihood rate algorithm. Judging from the frequency and chronological order of keywords, with the lapse of time, the hotspots of researchers have changed and gradually decreased. Relatively, “#0 insulin resistance,” “#3 overweight” and “#2 childhood obesity,” “#5 bariatric surgery” received relatively great attention.

### Visualization and analysis on co-citation reference

**Figure 7:**
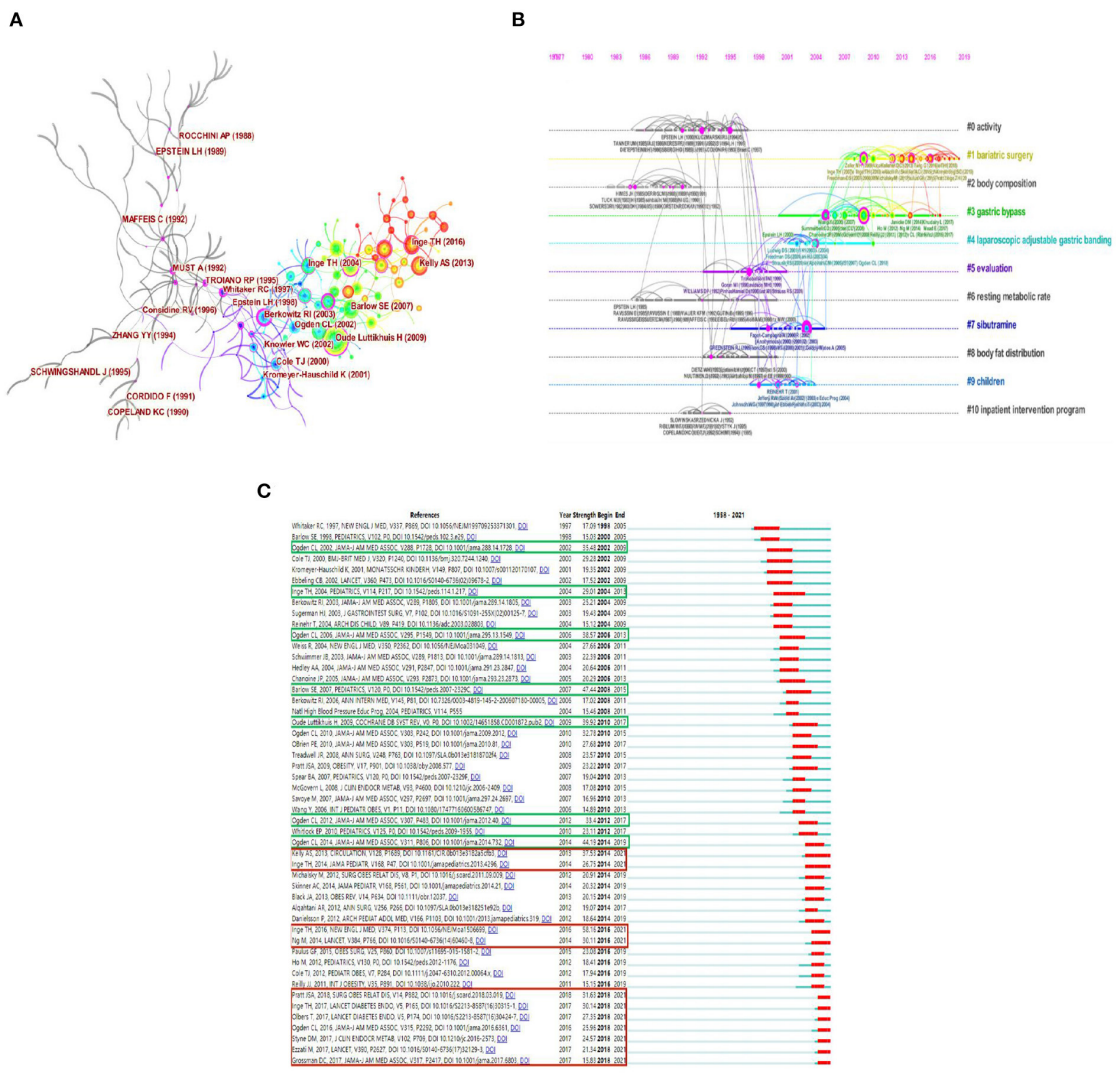
References co-citation network in the research of WLOCA **(A)**; Timeline map of co-citation clusters in the research of WLOCA **(B)**; Top 50 references with the strongest citation bursts in the research of WLOCA **(C)**.

Co-citation occurs when two studies from the early literature are both cited in a later study (61). Co-citation reference clustering can be regarded as a frontier field of academic research, and co-citation documents with strong burstiness can predict the frontier trend of future research in a certain field (62), especially the literature that has lasted so far (63). Figure 7A (Q = 0.7545 > 0.3, S = 0.9339 > 0.7) shows the top two documents with the highest frequency in each cluster. Relatively, the literature published after 2003 attracted attention of many researchers. Table 5 lists the top five co-citation articles ranked by frequency and centrality. The intervention treatment on childhood obesity has the highest co-citation frequency (36), while the randomized controlled trial of Sibutramine for the treatment and behavior therapy of adolescent obesity has the strongest centrality (30). These documents, as the knowledge basis for further research, play an important role in the study on WLOCA. Figure 7B shows the specific details of 11 frontier fields, relatively, “#1 bariatric surgery” is the current focus area of researchers. This study limited the minimum burst duration to 2 years and generated 108 bursts of references co-citation, and Figure 7C demonstrates the top 50 bursts. Among them, the study of Inge et al. (23) on health status in adolescents by bariatric surgery after 3 years had the highest burstiness of 58.16. The literature marked in Figure 7C are mainly on the topics of bariatric surgery, assessment and comprehensive treatment, and overweight/obesity prevalence survey.

**Table 5:** Top five references by co-citation frequency and centrality.

**Rank**	**Frequency**	**Co-citation reference**	**Centrality**	**Co-citation reference**
1	195	Oude et al. (36), *Cochrane Database Syst Rev*, V0, P0	0.74	Berkowitz et al. (30), *JAMA-J Am Med Assoc*, V289, P1805
2	161	Inge et al. (23), *New Engl J Med*, V374, P113	0.56	Chanoine et al. (31), *JAMA-J Am Med Assoc*, V293, P2873
3	138	Kelly et al. (64), *Circulation*, V128, P1689	0.47	Troiano et al. (65), *Arch Pediatr Adol Med*, V149, P1085
4	132	Barlow (66), *Pediatrics*, V120, P0	0.45	Must et al. (67), *New Engl J Med*, V327, P1350
5	111	Ogden et al. (68), *JAMA-J Am Med Assoc*, V311, P806	0.44	Whitaker et al. (69), *New Engl J Med*, V337, P869

## Discussion

The literature on WLOCA can be divided into three stages (Figure 2): the initiating stage (1958–1991), with little attention from the researchers and sporadic literature; the slowly developing stage (1992–2002), with a relatively gentle rise in literature and related fields beginning to attract the attention of researchers; and the rapidly developing stage (2003–2021), with fluctuations but significant overall increase. It's predicted that the global studies on WLOCA will further increase in the future, as a response to the epidemic of overweight and obesity in children and adolescents.

Institutional co-operation shows (Figure 3) that universities are the main forces in collaborative research, and children's hospitals are important co-operative members. Co-operative research is mainly concentrated in such developed countries as USA, Germany, England, and Australia. And the US has contributed the most. The types of co-operative institutions and cross-regional and cross-border co-operation are still relatively limited, which is not conducive to the global promotion and application of research findings. Co-operation between countries and institutions should be further strengthened, and the developing countries, in particular, should be given more concerns and supports. The co-operation between authors is not close enough (Figure 4), which shows that the low centrality of both high-yield authors and high-cited authors, and the core authors are few. However, the academic community represented by Thomas H. Inge, Thomas Reinehr, Todd M. Jenkins, Lian Tock, etc., as well as high co-citation, high centrality authors, and institutional co-operation information such as Epstein LH and Freedman DS can provide important clues for researchers to seek co-operation.

The journals that publish papers on WLOCA are quite extensive. Relatively, *Obesity, International Journal of Obesity, Obesity Surgery, Pediatrics*, and *Journal of the American Medical Association* are more preferred in this field, and researchers should keep track of these active journals to understand the latest research trends. Besides, the studies on WLOCA are characterized by being multidisciplinary and comprehensive (Figure 5B), mainly involving Medicine, Medical Clinical, Psychology, Education, Health, Sports, Neurology, Molecular, Biology, Immunology, etc. In addition, such disciplines as Nursing, Genetics, Environmental, Toxicology, Nutrition, Rehabilitation, Social are also important knowledge bases for WLOCA study.

Clustering of high-frequency keywords (Figure 6) shows that the 6 types of hotspots in the field of WLOCA study mainly focus on three aspects:

Firstly, assessment and pathophysiological mechanism (“#0 insulin resistance”). It mainly includes high-frequency keywords such as BMI, insulin resistance, metabolic syndrome, risk factor, leptin, insulin, and adiponectin. Studies show that BMI varies significantly with ages for children (70), measuring metabolic status by adopting a composite measures of BMI, waist-to-hip ratio, gender, genetic ethnicity, and metabolic markers may be of greater value in risk stratification of obesity (71). The etiology of obesity involves a variety of factors including genetic, epigenetic, environmental, dietary and lifestyle habits, and endocrine disorders (72), the molecular basis of which has not been fully elucidated (73). Adiponectin, leptin and plasminogen activator inhibitor 1 can be used as biomarkers to predict metabolic syndrome among adolescents (74). Leptin, resistin, interleukin-6, tumor necrosis factor-α, visfatin, and adiponectin play certain roles in energy homeostasis and glucose and lipid metabolism (75).

Secondly, comprehensive interventions (“#2 childhood obesity,” “#3 overweight”). It contains high-frequency keywords such as physical activity, exercise, program, family based treatment, behavior modification, prevention, and intervention. Numerous empirical studies on the time, intensity, and pattern of exercise, as well as in conjunction with dietary and behavioral changes have been conducted. Studies have shown that more physical activity and more intense exercise (76), and 155–180 min of moderate-to-high-intensity aerobic exercise per week (27), are beneficial in reducing body fat in overweight children. Combination of high-repetition resistance training, moderate aerobic exercise, and behavioral modification may be most effective in reducing body fat of overweight/obese children (77). A family therapy program that combines authoritative parenting styles with family function training can have a positive impact on the prevention and intervention of adolescent obesity (78), parents as the only modifiable factor can induce more behavioral changes in obese children and help them achieve more weight loss in the short term (26). Combined diet-behavior-physical activity interventions have both the short and long term beneficial effects for obese children (29), but the optimal combination remains unknown (79). Cognitive behavioral therapy (CBT) and motivational approaches are the most commonly used ones to carry out psychological therapy to achieve changes in dietary and exercise habits (80). Intensive interventions such as drugs or meal replacements should be combined with behavioral therapy (66). Olistat is currently the only approved drug for obese adolescents over 12 years old. It is limited in use with metformin, sibutramine and exenatide due to side effects of treatment (64, 72).

Finally, bariatric surgery (“#5 bariatric surgery”). It mainly includes keywords such as quality of life, randomized controlled trial, meta-analysis, y gastric bypass, laparoscopic sleeve gastrectomy. Inclusion criteria for bariatric surgery in obese adolescents are more stringent than adults and require a comprehensive preoperative assessment of potential health risks (81). Roux-en-Y gastric bypass surgery resulted in substantial and durable weight loss and cardiometabolic benefits for adolescents (82), LAGB significantly improves quality of life in most morbidly obese adolescents (24). LSG has become the first choice of patients with severe obesity in childhood worldwide, because it is a safe and effective solution to the the problem of obesity and its comorbidities (83–88). For long-term treatment success and healthy growth, post-operative micronutrient deficiencies, inadequate weight loss, weight rebound, psychosocial issues require further research (89, 90). In addition, the treatment of comorbidities such as cardiovascular disease caused by childhood obesity is also an important aspect (“#4 obesity”).

The timeline map of keywords co-occurrence (Figure 6B) shows the differences in research hotspots of scholars in different periods: From 1992 to 2002, the high-frequency keywords are relatively more diverse, including “physical activity,” “exercise,” “body composition,” “insulin,” “insulin resistance,” “risk factor,” “prevention,” “BMI,” “leptin,” etc. From 2003 to 2011, the research focus shifted to “metabolic syndrome,” “intervention,” “prevalence,” “bariatric surgery,” “quality of life” and so on. From 2012 to 2021, more attention were paid to “association,” “randomized controlled trial,” “y gastric bypass,” “meta-analysis,” “laparoscopic sleeve gastrectomy.” On the whole, the research focus has gradually shifted from lifestyle intervention and pathophysiological mechanism to clinical surgical applications in severely obese adolescents. This research trend shows that interventions mainly based on physical activity and diet do not seem to be effective in addressing all obesity problems, and also reflects the complexity of overweight/obesity in children and adolescents. Therefore, it is necessary to further explore the influencing factors.

Obesity is not caused by a single factor, but the result of interactions between environmental, behavioral, developmental, biological, and genetic factors (91), gut microbiota, epigenetics, and intrauterine and generational effects are also recently identified contributing factors (92, 93). Specifically, due to infant and toddler feeding patterns, excessive parental dietary restrictions, peer interactions, mental health issues (depression, social isolation, bullying, stigma), frequent electronic device use, lack of sleep, sedentary, individual neurological Differences in hormonal regulation structure, gene-environment interaction, adolescent growth and development, etc., coupled with such social factors as school and family environment, community and socioeconomic resources, make it difficult for a single intervention and treatment plan to achieve the ideal long-term weight loss effect for children and adolescents. At the same time, the metabolic syndrome and related comorbidities, such as non-alcoholic fatty liver disease (NAFLD), polycystic ovary syndrome (PCOS), and OSAS, which are often accompanied by obesity in children and adolescents, bring more challenges to appropriate weight loss programs. Due to multiple additive effects, a combination of drugs and bariatric surgery may eventually have to be selected. Notably, strict dietary control (low-calorie diet) can be effective in reducing the risk of obesity-related comorbidities, such as metabolic syndrome, T2DM, but caution is needed for serious consequences of eating disorders (91). A systematic review shows that tonsillectomy can improve the behavior cognition and quality of life of children with OSAS (94). In morbidly or severely obese adolescents, the improvement of comorbidities varied by bariatric surgery (90, 95), while LSG and LAGB showed significant effect in relieving the symptoms of T2DM, dyslipidemia, bronchial asthma, NAFLD, and OSAS (96, 97), indicating certain advantages. More effective bariatric surgery or alternatives are yet to be discovered in practice, and multidisciplinary research is necessary as well.

At the same time, we should also be aware that there must be some differences in the intervention programs for overweight/obese children and adolescents under multiple causes, which are also reflected in the different stages of development of overweight/obesity. It is necessary to strengthen the monitoring of possible problems and adopt targeted combination programs to improve the effectiveness of intervention treatment. However, lifestyle interventions (physical activity, diet) are always an integral part of the prevention and treatment of overweight/obesity in children and adolescents. It should be emphasized that researchers should establish a good ethical awareness, maintain communication with the ethics committee and strictly implement management norms. The research or intervention and treatment process should put the protection of the rights and interests of the subjects in the first place.

Combined with the strong burstiness literature (Figure 7) and the research hotspots above and their development, it can be found that there are certain trends for future research: Investigation into the prevalence of overweight/obesity in children and adolescents will remain an important part of literature, while prospective studies on bariatric surgery will be more common. Besides, the study on pathophysiological mechanism will comprehensively take physiological, genetic, psychological and environmental factors into consideration. And Neuro-endocrinology, Molecular Biology, Immunology, Psychiatry and Social Ecology will be combined for interdisciplinary research. In addition, to cope with the worsening problem of overweight/obesity in children and adolescents, how to effectively improve health literacy to ensure “active health” will be further strengthened, while the school-age period, and prenatal, infant and preschool will be important stages of prevention research. Meanwhile, the studies on WLOCA will be more refined and differentiated, and characterized by multidisciplinary combination, classification and phasing, in light of different ages, genders, cognitive development, obesity levels, ethnic groups, and cultural backgrounds. The “dose-effect” comparative study of intervention combination therapy for behavior change will further increase. New drug development and combination therapy based on adult treatment will continue to advance. Studies on laparoscopic sleeve gastrectomy will be on a rise due to its popularity in the treatment of severely obese adolescents, including possible nutritional deficiencies, weight regain, and psychological problems after surgery. However, more effective medical and surgical methods in the future are the direction of efforts, such as therapeutic interventions at different gut-liver axis levels. It is noteworthy that the COVID-19 pandemic has significant impacts on every aspects of human life, so the psychosocial and individualized interventions for overweight/obese children and adolescents should also be given attention. What's more, promoting the overall effect of schools, families, hospitals and communities in obesity management of children and adolescents also plays an important role in alleviating the problem of overweight/obesity in children and adolescents, which requires the joint efforts of various stakeholders, and supports from policies and research funding. Therefore, studies from such perspectives as sociology, politics and economics will also interest researchers.

### Limitation and future research

The limitation of this study is that databases beyond WoSCC, books, non-English and unpublished government documents, dissertations, conference papers, scientific and technical reports, and other literature were not considered, which may lead to the consequence that the research findings may not be a panorama sketch of the field. What's more, the main focus of the interpretation is on the high frequency, high centrality, and high burst nodes, thus some other details may be overlooked. Therefore, future studies should extend the retrieval and read more relevant literature. Meanwhile, it is recommended that researchers should take the comprehensiveness and consistency of literature retrieved into consideration so as to ensure the reliability of the research results.

## Conclusions

Research on WLOCA has seen positive developments in all aspects. Compared with general retrospective papers, the contribution of this study lies in its revealing of institutions, countries/regions, active journals, disciplinary interactions, core authors and their academic communities, and references that play an important role in the studied of WLOCA with rich maps and detailed tables. It intuitively presents the current research hotspots and developments, and predicts future research trends. It can provide valuable information for relevant researchers to quickly understand the research status in this field, find co-operation, track research hotspots and frontier trends.

## Data availability statement

The original contributions presented in the study are included in the article/supplementary material, further inquiries can be directed to the corresponding author/s.

## Author contributions

GS: conceptualization, methodology, formal analysis, data curation, writing—original draft preparation, visualization, and funding acquisition. LL: validation, formal analysis, data curation, writing—review and editing, and supervision. XZ: methodology, validation, writing—review and editing, and supervision. All authors have read and agreed to the published version of the manuscript.

## Funding

This work was supported by the Humanities and Social Sciences Project of Hubei Provincial Education Department of China under Grant number 20Q038.

## Conflict of interest

The authors declare that the research was conducted in the absence of any commercial or financial relationships that could be construed as a potential conflict of interest.

## Publisher's note

All claims expressed in this article are solely those of the authors and do not necessarily represent those of their affiliated organizations, or those of the publisher, the editors and the reviewers. Any product that may be evaluated in this article, or claim that may be made by its manufacturer, is not guaranteed or endorsed by the publisher.

## References

[1] Ng M Fleming T Robinson M Thomson B Graetz N Margono C. Global, regional, and national prevalence of overweight and obesity in children and adults during 1980–2013: a systematic analysis for the Global Burden of Disease Study 2013. Lancet. (2014) 384:766–81. 10.1016/S0140-6736(14)60460-8 24880830 PMC4624264

[2] Ogden CL Carrol MD Flegal KM. High body mass index for age among US children and adolescents, 2003–2006. J Am Med Assoc. (2008) 299:2401–5. 10.1001/jama.299.20.2401 18505949

[3] NCD-RisC. Worldwide trends in body-mass index, underweight, overweight, and obesity from 1975 to 2016: a pooled analysis of 2416 population-based measurement studies in 128.9 million children, adolescents, and adults. Lancet. (2017) 390:2627–42. 10.1016/S0140-6736(17)32129-3 29029897 PMC5735219

[4] World Health Organization. Obesity and Overweight. (2021). Available online at: https://www.who.int/zh/news-room/fact-sheets/detail/obesity-and-overweight (accessed June 3, 2022).

[5] Wen J Zhu LJ Ji CB. Changes in weight and height among Chinese preschool children during COVID-19 school closures. Int J Obes. (2021) 45:2269–73. 10.1038/s41366-021-00912-4 34267325 PMC8281806

[6] Kim ES Kwon Y Choe YH Kim MJ. COVID-19-related school closing aggravate obesity and glucose intolerance in pediatric patients with obesity. Sci Rep. (2021) 11:5494. 10.1038/s41598-021-84766-w 33750841 PMC7943757

[7] Lobstein T Baur L Uauy R. Obesity in children and young people: a crisis in public health. Obes Rev. (2004) 5:4–104. 10.1111/j.1467-789X.2004.00133.x 15096099

[8] Park MH Falconer C Viner RM Kinra S. The impact of childhood obesity on morbidity and mortality in adulthood: a systematic review. Obes Rev. (2012) 13:985–1000. 10.1111/j.1467-789X.2012.01015.x 22731928

[9] Singh AS Mulder C Twisk JWR van Mechelen W Chinapaw MJM. Tracking of childhood overweight into adulthood: a systematic review of the literature. Obes Rev. (2008) 9:474–88. 10.1111/j.1467-789X.2008.00475.x 18331423

[10] Quek YH Tam WWS Zhang MWB Ho RCM. Exploring the association between childhood and adolescent obesity and depression: a meta-analysis. Obes Rev. (2017) 18:742–54. 10.1111/obr.12535 28401646

[11] Caird J Kavanagh J O'Mara-Eves A Oliver K Oliver S Stansfield C. Does being overweight impede academic attainment? A systematic review. Health Educ J. (2014) 73:497–521. 10.1177/0017896913489289

[12] Daniels SR Arnett DK Eckel RH Gidding SS Hayman LL Kumanyika S. Overweight in children and adolescents—pathophysiology, consequences, prevention, and treatment. Circulation. (2005) 111:1999–2012. 10.1161/01.CIR.0000161369.71722.10 15837955

[13] De Leonibus C Marcovecchio ML Chiarelli F. Update on statural growth and pubertal development in obese children. Pediatr Rep. (2012) 4:e35. 10.4081/pr.2012.e35 23355935 PMC3555205

[14] Gurnani M Birken C Hamilton J. Childhood obesity: causes, consequences, and management. Pediatr Clin North Am. (2015) 62:821–40. 10.1016/j.pcl.2015.04.001 26210619

[15] Marcus CL Brooks LJ Ward SD Draper KA Gozal D Halbower AC. Diagnosis and management of childhood obstructive sleep apnea syndrome. Pediatrics. (2012) 130:e714–55. 10.1542/peds.2012-1672 22926176

[16] Pollicina I Maniaci A Lechien JR Iannella G Vicini C Cammaroto G. Neurocognitive performance improvement after obstructive sleep apnea treatment: state of the art. Behav Sci. (2021) 11:180. 10.3390/bs11120180 34940115 PMC8698492

[17] Cote AT Harris KC Panagiotopoulos C Sandor GGS Devlin AM. Childhood obesity and cardiovascular dysfunction. J Am Coll Cardiol. (2013) 62:1309–19. 10.1016/j.jacc.2013.07.042 23954339

[18] Bhattacharjee R Kheirandish-Gozal L Pillar G Gozal D. Cardiovascular complications of obstructive sleep apnea syndrome: evidence from children. Prog Cardiovasc Dis. (2009) 51:416–33. 10.1016/j.pcad.2008.03.002 19249448

[19] Bibbins-Domingo K Coxson P Pletcher MJ Lightwood J Goldman L. Adolescent overweight and future adult coronary heart disease. N Engl J Med. (2007) 357:2371–9. 10.1056/NEJMsa073166 18057339

[20] Styne DM Arslanian SA Connor EL Farooqi IS Murad MH Silverstein JH. Pediatric obesity-assessment, treatment, and prevention: an endocrine society clinical practice guideline. J Clin Endocr Metab. (2017) 102:709–57. 10.1210/jc.2016-2573 28359099 PMC6283429

[21] Reinehr T Kleber M Toschke AM. Lifestyle intervention in obese children is associated with a decrease of the metabolic syndrome prevalence. Atherosclerosis. (2009) 207:174–80. 10.1016/j.atherosclerosis.2009.03.041 19442975

[22] Grulich-Henn J Lichtenstein S Horster F Hoffmann GF Nawroth PP Hamann A. Moderate weight reduction in an outpatient obesity intervention program significantly reduces insulin resistance and risk factors for cardiovascular disease in severely obese adolescents. Int J Endocrinol. (2011) 2011:541021. 10.1155/2011/541021 21904547 PMC3166723

[23] Inge TH Courcoulas AP Jenkins TM Michalsky MP Helmrath MA Brandt ML. Weight loss and health status 3 years after bariatric surgery in adolescents. New Engl J Med. (2016) 374:113–23. 10.1056/NEJMoa1506699 26544725 PMC4810437

[24] Silberhumer GR Miller K Kriwanek S Widhalm K Pump A Prager G. Laparoscopic adjustable gastric banding in adolescents: the Austrian experience. Obes Surg. (2006) 16:1062–7. 10.1381/096089206778026262 16901361

[25] Silva DFO Sena-Evangelista KCM Lyra CO Pedrosa LFC Arrais RF Lima SCVC. Motivations for weight loss in adolescents with overweight and obesity: a systematic review. BMC Pediatr. (2018) 18:364. 10.1186/s12887-018-1333-2 30463551 PMC6247735

[26] Golan M Fainaru M Weizman A. Role of behavior modification in the treatment of childhood obesity with the parents as the exclusive agents of change. Int J Obesity. (1998) 22:1217–24. 10.1038/sj.ijo.0800749 9877257

[27] Atlantis E Barnes EH Singh MAF. Efficacy of exercise for treating overweight in children and adolescents: a systematic review. Int J Obesity. (2006) 30:1027–40. 10.1038/sj.ijo.0803286 16534526

[28] Bulbul S. Exercise in the treatment of childhood obesity. Turk Pediatri Ars. (2020) 55:2–10. 10.14744/TurkPediatriArs.2019.60430 32231444 PMC7096559

[29] Nemet D Barkan S Epstein Y Friedland O Kowen G Eliakim A. Short- and long-term beneficial effects of a combined dietary-behavioral-physical activity intervention for the treatment of childhood obesity. Pediatrics. (2005) 115:e443–9. 10.1542/peds.2004-2172 15805347

[30] Berkowitz RI Wadden TA Tershakovec AM Cronquist JL. Behavior therapy and sibutramine for the treatment of adolescent obesity—a randomized controlled trial. J Am Med Assoc. (2003) 289:1805–12. 10.1001/jama.289.14.1805 12684359

[31] Chanoine JP Hampl S Jensen C Boldrin M Hauptman J. Effect of orlistat on weight and body composition in obese adolescents—a randomized controlled trial. J Am Med Assoc. (2005) 293:2873–83. 10.1001/jama.293.23.2873 15956632

[32] Pratt JSA Lenders CM Dionne EA Hoppin AG Hsu GLK Inge TH. Best practice updates for pediatric/adolescent weight loss surgery. Obesity. (2009) 17:901–10. 10.1038/oby.2008.577 19396070 PMC3235623

[33] Inge TH Zeller MH Jenkins TM Helmrath M Brandt ML Michalsky MP. Perioperative outcomes of adolescents undergoing bariatric surgery the teen-longitudinal assessment of bariatric surgery (Teen-LABS) study. JAMA Pediatr. (2014) 168:47–53. 10.1001/jamapediatrics.2013.4296 24189578 PMC4060250

[34] Pedroso FE Angriman F Endo A Dasenbrock H Storino A Castillo R. Weight loss after bariatric surgery in obese adolescents: a systematic review and meta-analysis. Surg Obes Relat Dis. (2018) 14:413422. 10.1016/j.soard.2017.10.003 29248351

[35] Kobes A Kretschmer T Timmerman G Schreuder P. Interventions aimed at preventing and reducing overweight/obesity among children and adolescents: a meta-synthesis. Obes Rev. (2018) 19:1065–79. 10.1111/obr.12688 29671938

[36] Oude Luttikhuis H Baur L Jansen H Shrewsbury VA O'Malley C Stolk RP. Interventions for treating obesity in children. Cochrane Database Syst Rev. (2009) CD001872. 10.1002/14651858.CD001872.pub2 19160202

[37] Chen CM. CiteSpace II: detecting and visualizing emerging trends and transient patterns in scientific literature. J Assoc Inf Sci Tech. (2006) 57:359–77. 10.1002/asi.20317

[38] Chen CM Chen Y Horowitz M Hou HY Liu ZY Pellegrino D. Towards an explanatory and computational theory of scientific discovery. J Informetr. (2009) 3:191–209. 10.1016/j.joi.2009.03.004

[39] Chen CM Hu ZG Liu SB Tseng H. Emerging trends in regenerative medicine: a scientometric analysis in CiteSpace. Expert Opin Biol Th. (2012) 12:593–608. 10.1517/14712598.2012.674507 22443895

[40] Yi F Yang P Sheng H. Tracing the scientific outputs in the field of Ebola research based on publications in the web of science. BMC Res Notes. (2016) 9:221. 10.1186/s13104-016-2026-2 27083891 PMC4832479

[41] Chen DY Zhang G Wang JH Chen SL Wang JX Nie H. Mapping trends in moyamoya angiopathy research: a 10-year bibliometric and visualization-based analyzes of the web of science core collection (WoSCC). Front Psychol. (2021) 12:637310. 10.3389/fneur.2021.637310 33737903 PMC7960774

[42] Chinchilla-Rodriguez Z Zacca-Gonzalez G Vargas-Quesada B Moya-Anegon F. Latin American scientific output in public health: combined analysis using bibliometric, socioeconomic and health indicators. Scientometrics. (2015) 102:609628. 10.1007/s11192-014-1349-9

[43] Wang MX Liu P Zhang R Li Z Li X. A scientometric analysis of global health research. Int J Environ Res Public Health. (2020) 17:2963. 10.3390/ijerph17082963 32344668 PMC7215720

[44] Sun JF Zhou ZC Huang J Li GX. A bibliometric analysis of the impacts of air pollution on children. Int J Environ Res Public Health. (2020) 17:1277. 10.3390/ijerph17041277 32079218 PMC7068507

[45] Guo F Li FX Lv W Li L Duffy VG. Bibliometric analysis of affective computing researches during 1999–2018. Int J Hum-Comput Int. (2019) 36:801–14. 10.1080/10447318.2019.1688985

[46] Tran BX Latkin CA Vu GT Nguyen HLT Nghiem S Tan MX. The current research landscape of the application of artificial intelligence in managing cerebrovascular and heart diseases: a bibliometric and content analysis. Int J Environ Res Public Health. (2019) 16:2699. 10.3390/ijerph16152699 31362340 PMC6696240

[47] Xiang CY Wang Y Liu HW. A scientometrics review on non-point source pollution research. Ecol Eng. (2017) 99:400–8. 10.1016/j.ecoleng.2016.11.02834365829

[48] Xu MD Williams PJ Gu JJ Zhang H. Hotspots and trends of technology education in the international journal of technology and design education: 2000–2018. Int J Technol Des Ed. (2020) 30:207–24. 10.1007/s10798-019-09508-6

[49] You YW Min LZ Tang MH Chen YQ Ma XD. Bibliometric evaluation of global Tai Chi research from 1980–2020. Int J Environ Res Public Health. (2021) 18:6150. 10.3390/ijerph18116150 34200236 PMC8201343

[50] Chadegani AA Salehi H Yunus MM Farhadi H Fooladi M Farhadi M. A comparison between two main academic literature collections: web of science and scopus databases. Asian Social Sci. (2013) 9:18–26. 10.5539/ass.v9n5p18

[51] Falagas ME Pitsouni EI Malietzis GA Pappas G. Comparison of pubmed, scopus, web of science, and google scholar: strengths and weaknesses. FASEB J. (2008) 22:338–42. 10.1096/fj.07-9492LSF 17884971

[52] Liang YD Li Y Zhao J Wang XY Zhu HZ Chen XH. Study of acupuncture for low back pain in recent 20 years: a bibliometric analysis *via* CiteSpace. J Pain Res. (2017) 10:951–64. 10.2147/JPR.S132808 28479858 PMC5411170

[53] Yan WW Zheng KY Weng LM Chen CC Kiartivich S Jiang X. Bibliometric evaluation of 2000–2019 publications on functional near-infrared spectroscopy. Neuroimage. (2020) 220:117121. 10.1016/j.neuroimage.2020.117121 32619709

[54] Gonzalez J Garijo I Sanchez A. Organ trafficking and migration: a bibliometric analysis of an untold story. Int J Environ Res Public Health. (2020) 17:3204. 10.3390/ijerph17093204 32380680 PMC7246946

[55] Li KL Weng LM Wang XQ. The state of music therapy studies in the past 20 years: a bibliometric analysis. Front Psychol. (2021) 12:697726. 10.3389/fpsyg.2021.697726 34177744 PMC8222602

[56] Chen BY Shin S. Bibliometric analysis on research trend of accidental falls in older adults by using Citespace-focused on web of science core collection (2010–2020). Int J Environ Res Public Health. (2021) 18:1663. 10.3390/ijerph18041663 33572483 PMC7916410

[57] Chen Y Chen CM Hu ZG Wang XW. Principles and Applications of Analyzing a Citation Space. Beijing: Science Press (2015). p. 43.

[58] Chen Y Chen CM Hu ZG Wang XW. Principles and Applications of Analyzing a Citation Space. Beijing: Science Press (2015). p. 82–4.

[59] CHen CM Leydesdorff L. Patterns of connections and movements in dual-map overlays: a new method of publication portolio analysis. J Am Soc Inf Sci Tec. (2014) 65:334–51. 10.1002/asi.22968

[60] Wang ZH Zhao YD Wang B. A bibliometric analysis of climate change adaptation based on massive research literature data. J Clean Prod. (2018) 199:1072–82. 10.1016/j.jclepro.2018.06.183

[61] Small H. Co-citation in the scientific literature: a new measure of the relationship between two documents. J Am Soc Inf Sci. (1973) 24:265–9. 10.1002/asi.4630240406

[62] Fitzpatrick RB. Essential science indicators. Med Ref Serv Q. (2005) 24:67–78. 10.1300/J115v24n04_05 16203702

[63] Fu LP Sun ZH He LP Liu F Jing XL. Global long-term care research: a scientometric review. Int J Environ Res Public Health. (2019) 16:2077. 10.3390/ijerph16122077 31212782 PMC6616636

[64] Kelly AS Barlow SE Rao G Inge TH Hayman LL Steinberger J. Severe obesity in children and adolescents: identification, associated health risks, and treatment approaches a scientific statement from the American Heart Association. Circulation. (2013) 128:1689–712. 10.1161/CIR.0b013e3182a5cfb3 24016455

[65] Troiano RP Flegal KM Kuczmarski RJ Campbell SM Johnson CL. Overweight prevalence and trends for children and adolescents. The National Health and Nutrition Examination Surveys, 1963 to 1991. Arch Pediatr Adolesc Med. (1995) 149:1085–91. 10.1001/archpedi.1995.02170230039005 7550810

[66] Barlow SE. Expert committee recommendations regarding the prevention, assessment, and treatment of child and adolescent overweight and obesity: summary report. Pediatrics. (2007) 120:S164–92. 10.1542/peds.2007-2329C 18055651

[67] Must A Jacques PF Dallal GE Bajema CJ Dietz WH. Long-term morbidity and mortality of overweight adolescents. A follow-up of the Harvard Growth Study of 1922 to 1935. New Engl J Med. (1992) 327:1350–5. 10.1056/NEJM199211053271904 1406836

[68] Ogden CL Carroll MD Kit BK Flegal KM. Prevalence of childhood and adult obesity in the United States, 2011–2012. J Am Med Assoc. (2014) 311:806–14. 10.1001/jama.2014.732 24570244 PMC4770258

[69] Whitaker RC Wright JA Pepe MS Seidel KD Dietz WH. Predicting obesity in young adulthood from childhood and parental obesity. New Engl J Med. (1997) 337:869–73. 10.1056/NEJM199709253371301 9302300

[70] Cole TJ Bellizzi MC Flegal KM Dietz WH. Establishing a standard definition for child overweight and obesity worldwide: international survey. BMJ-Brit Med J. (2000) 320:1240–3. 10.1136/bmj.320.7244.1240 10797032 PMC27365

[71] Nguyen N Champion JK Ponce J Quebbemann B Patterson E Pham B. A review of unmet needs in obesity management. Obes Surg. (2012) 22:956–66. 10.1007/s11695-012-0634-z 22438220

[72] Catoira N Nagel M Di Girolamo G Gonzalez CD. Pharmacological treatment of obesity in children and adolescents: current status and perspectives. Expert Opin Pharmaco. (2010) 11:2973–83. 10.1517/14656566.2010.512005 20958104

[73] Crocker MK Yanovski JA. Pediatric obesity: etiology and treatment. Endocrinol Metab Clin North Am. (2009) 38:525–48. 10.1016/j.ecl.2009.06.007 19717003 PMC2736391

[74] Gonzalez M Bibiloni MD Pons A Llompart I Tur JA. Inflammatory markers and metabolic syndrome among adolescents. Eur J Clin Nutr. (2012) 66:1141–5. 10.1038/ejcn.2012.112 22909576

[75] Pyrzak B Ruminska M Popko K Demkow U. Adiponectin as a biomarker of the metabolic syndrome in children and adolescents. Eur J Med Res. (2010) 15:147–51. 10.1186/2047-783X-15-S2-147 21147643 PMC4360280

[76] Boutelle KN Hannan PJ Neumark-Sztainer D Himes JH. Identification and correlates of weight loss in adolescents in a national sample. Obesity. (2007) 15:473–82. 10.1038/oby.2007.501 17299121

[77] Bennett B Sothern MS. Diet, exercise, behavior: the promise and limits of lifestyle change. Semin Pediatr Surg. (2009) 18:152–8. 10.1053/j.sempedsurg.2009.04.005 19573757 PMC2744116

[78] Kitzman-Ulrich H Wilson DK St George SM Lawman H Segal M Fairchild A. The integration of a family systems approach for understanding youth obesity, physical activity, and dietary programs. Clin Child Fam Psych. (2010) 13:231–53. 10.1007/s10567-010-0073-0 20689989 PMC3293190

[79] Owens S Galloway R. Childhood obesity and the metabolic syndrome. Curr Atheroscler Rep. (2014) 16:436. 10.1007/s11883-014-0436-y 25037582

[80] Boff RD Liboni RPA Batista IPD de Souza LH Oliveira MD. Weight loss interventions for overweight and obese adolescents: a systematic review. Eat Weight Disord-St. (2017) 22:211–29. 10.1007/s40519-016-0309-1 27542161

[81] Di Lorenzo N Antoniou SA Batterham RL Busetto L Godoroja D Iossa A. Clinical practice guidelines of the European Association for Endoscopic Surgery (EAES) on bariatric surgery: update 2020 endorsed by IFSO-EC, EASO, and ESPCOP. Surg Endosc. (2020) 34:2332–58. 10.1007/s00464-020-07555-y 32328827 PMC7214495

[82] Inge TH Jenkins TM Xanthakos SA Dixon JB Daniels SR Zeller MH. Long-term outcomes of bariatric surgery in adolescents with severe obesity (FABS-5+): a prospective follow-up analysis. Lancet Diabetes Endo. (2017) 5:165–73. 10.1016/S2213-8587(16)30315-1 28065736 PMC8282411

[83] Arman GA. Long-term (11+ years) outcomes in weight, patient satisfaction, comorbidities, and gastroesophageal reflux treatment after laparoscopic sleeve gastrectomy. Surg Obes Relat Dis. (2016) 12:1778–86. 10.1016/j.soard.2016.01.013 27178613

[84] Durkin N Desai AP. What is the evidence for paediatric/adolescent bariatric surgery? Curr Obes Rep. (2017) 6:278–85. 10.1007/s13679-017-0277-4 28815416 PMC5585991

[85] Marnicola G Gallo C Hassan C Raffaelli M Costamagna G Bove V. Laparoscopic sleeve gastrectomy vs endoscopic sleeve gastroplasty: a systematic review and meta-analysis. Endosc Int Open. (2021) 9:E87–95. 10.1055/a-1300-1085 33403240 PMC7775813

[86] Ejaz A Patel P Gonzalez-Heredia R Holterman M Elli EF Kanard R. Laparoscopic sleeve gastrectomy as first-line surgical treatment for morbid obesity among adolescents. J Pediatric Surg. (2017) 52:544–8. 10.1016/j.jpedsurg.2016.08.023 27637140

[87] Tuna T Espinheira MD Vasconcelos C Preto J Campos JM. Laparoscopic sleeve gastrectomy in morbidly obese adolescents: initial experience of a pediatric multidisciplinary unit. Arch Pediatr. (2020) 27:310–4. 10.1016/j.arcped.2020.06.003 32651142

[88] Paulus GF de Vaan LEG Verdam FJ Bouvy ND Ambergen TAW van Heurn LWE. Bariatric surgery in morbidly obese adolescents: a systematic review and meta-analysis. Obes Surg. (2015) 25:860–78. 10.1007/s11695-015-1581-2 25697125 PMC4428750

[89] Herget S Rudolph A Hilbert A Bluher S. Psychosocial status and mental health in adolescents before and after bariatric surgery: a systematic literature review. Obes Facts. (2014) 7:233–45. 10.1159/000365793 25059420 PMC5644788

[90] Calcaterra V Cena H Pelizzo G Porri D Regalbuto C Vinci F. Bariatric surgery in adolescents: to do or not to do? Children. (2021) 8:453. 10.3390/children8060453 34072065 PMC8204230

[91] Kansra AR Lakkunarajah S Jay MS. Childhood and adolescent obesity: a review. Front Pediatr. (2021) 8:581461. 10.3389/fped.2020.581461 33511092 PMC7835259

[92] Rinninella E Raoul P Cintoni M Fransceschi F Miggiano GAD Gasbarrini A. What is the healthy gut microbiota composition? A changing ecosystem across age, environment, diet, and diseases. Microorganisms. (2019) 7:14. 10.3390/microorganisms7010014 30634578 PMC6351938

[93] Indrio F Martini S Francavilla R Corvaglia L Cristofori F Mastrolia SA. Epigenetic matters: the link between early nutrition, microbiome, and long-term health development. Front Pediatr. (2017) 5:178. 10.3389/fped.2017.00178 28879172 PMC5572264

[94] Di Mauro P Cocuzza S Maniaci A Ferlito S Rasa D Anzivino R. The effect of adenotonsillectomy on children's behavior and cognitive performance with obstructive sleep apnea syndrome: state of the art. Children. (2021) 8:921. 10.3390/children8100921 34682186 PMC8535044

[95] Black JA White B Viner RM Simmons RK. Bariatric surgery for obese children and adolescents: a systematic review and meta-analysis. Obes Rev. (2013) 14:634–44. 10.1111/obr.12037 23577666

[96] Messiah SE Lopez-Mitnik G Winegar D Sherif B Arheart KL Reichard KW. Changes in weight and co-morbidities among adolescents undergoing bariatric surgery: 1-year results from the bariatric outcomes longitudinal database. Surg Obes Relat Dis. (2013) 9:503–13. 10.1016/j.soard.2012.03.007 22542199 PMC3416929

[97] Alqahtani AR Elahmedi MO Al Qahtani A. Co-morbidity resolution in morbidly obese children and adolescents undergoing sleeve gastrectomy. Surg Obes Relat Dis. (2014) 10:842–50. 10.1016/j.soard.2014.01.020 25439000

